# Pensar fora da caixa: uma outra ciência é urgente

**DOI:** 10.1590/S0104-59702025000100005

**Published:** 2025-02-14

**Authors:** Glaucia Guimarães Pereira

**Affiliations:** i Doutoranda, Centro de Desenvolvimento Sustentável/Universidade de Brasília. Brasília – DF – Brasil. glauciagp23@gmail.com

Isabelle Stengers, filósofa belga nascida em 1949, pensa e conecta a ciência com perspectivas abrangentes e inovadoras. Sua carreira é marcada por estudos sobre a ciência e suas interações com a sociedade. Formada inicialmente em química, os seus interesses migraram para a filosofia. Foi professora no departamento de Filosofia, Ética e Ciências das Religiões e da Laicidade da Universidade Livre de Bruxelas.

Originalmente publicado em francês, em 2013, *Uma outra ciência é possível: manifesto por uma desaceleração das ciências* é enriquecido por um prefácio exclusivo para a edição brasileira. Nele, a autora pontua a relevância permanente de suas ideias e nos convida a refletir sobre a atualidade do debate, reafirmando que é necessária e possível uma nova forma de fazer ciência, pois a ciência atual é frequentemente conduzida sob pressões de tempo e eficácia que comprometem a integridade ética e a relevância social das pesquisas.

Stengers (2023) questiona a definição de pares, apontando que as trocas e leituras da produção científica são feitas cada vez mais por grupos superespecializados. Questiona também quais vozes são amplificadas ou silenciadas nesse processo de publicações especializadas e que visam a métricas de impacto.

Ela defende a revisão das normas estabelecidas de validação do conhecimento, e critica a rigidez dos métodos das ciências “duras”, que frequentemente excluem questões consideradas “não científicas”, ignorando perspectivas que poderiam enriquecer o entendimento e a aplicação da ciência. Essa visão é coerente com Ailton Krenak (2020), quando o autor aborda o papel do conhecimento em uma perspectiva mais ampla e integradora, sugerindo que nossa visão do mundo, dos sonhos e da natureza deve ir além dos limites rígidos impostos pela ciência ocidental.

Para Stengers, desacelerar significa prestar atenção às questões que realmente importam, aquelas cujas respostas lançariam luz sobre as mazelas sociais ou as mudanças climáticas. Significa parar de categorizar questões como não científicas só porque os métodos consolidados de um campo especializado não podem respondê-las. Ela sugere que a ciência deve se abrir para um diálogo mais amplo com a sociedade, incluindo vozes que tradicionalmente não são consideradas parte do discurso científico.

A autora desafia concepções tradicionais de objetividade e racionalidade científicas, apontando para uma visão mais inclusiva sobre fazer ciência. Ela se reporta principalmente aos integrantes da comunidade acadêmica, fazendo apelos por uma desaceleração das ciências. Se a leitura não exigisse familiaridade com as práticas acadêmicas e com a estrutura de gestão científica, eu diria que o livro é para ser lido por qualquer pessoa interessada em saber de que forma vamos enfrentar, como sociedade fora da academia, as crises sociais e ambientais contemporâneas.

A leitura, porém, é exigente, e o livro é particularmente valioso para as pessoas que já têm familiaridade com debates científicos e filosóficos, sendo particularmente interessante para quem atua em instituições e temas interdisciplinares. O esforço para compreender as ideias de Stengers pode ser recompensador, proporcionando reflexões sobre como a ciência pode interagir melhor com a sociedade. Conforme avalia também Sheila Jasanoff (2021), o público, frequentemente visto como receptor passivo do conhecimento científico, deveria ser mais amplamente envolvido e informado sobre as metodologias científicas, as escolhas feitas em nome da objetividade e as implicações dessas escolhas.

Iniciei a leitura supondo que a desaceleração proposta por Stengers se concentrasse, principalmente, nas ciências rápidas e duras. No entanto, ela aborda como as ciências sociais também precisam desacelerar, pois foram contaminadas pelos valores das ciências camerais, isto é, aquelas ligadas à administração pública. Essa perspectiva amplia o debate para além do esperado, sugerindo uma revisão crítica de como as práticas e pressões das ciências aplicadas afetam campos tradicionalmente menos focados na produção acelerada de resultados, como as ciências sociais.

Sendo assim, é preciso pensar “fora da caixa”, fora das barreiras das disciplinas. Isso exige mudanças amplas: compreender a aplicabilidade e os limites de métodos, entender a influência da dinâmica de mercado na produção científica e incorporar saberes para além da academia. Stengers sugere que a ciência, em vez de se apresentar como um bastião de objetividade e racionalidade, sendo, por isso, superior, deveria se engajar em um diálogo respeitoso e civilizado com outras formas de conhecimento, reconhecendo a sua validade. Essa ciência lenta enriquece a qualidade do conhecimento produzido, ao incluir diversas perspectivas que vêm sendo marginalizadas.

*Uma outra ciência é possível* é leitura essencial para quem vive nas ciências e busca uma perspectiva renovadora sobre as práticas científicas contemporâneas. Apresenta ao público brasileiro uma obra de relevância internacional e adiciona uma camada de contextualização crítica ao papel da ciência no enfrentamento de crises globais. Stengers nos convida a repensar como a ciência pode e deve evoluir em resposta às necessidades urgentes de nossa sociedade. Portanto, recomendo sua leitura para todos os pesquisadores e pensadores engajados em pesquisas científicas e na produção de textos científicos.
